# Biochar Supplementation Effects on Fresh Goat Meat and Carcass Characteristics

**DOI:** 10.3390/ani16071074

**Published:** 2026-04-01

**Authors:** Savannah L. Douglas, Nina E. Gilmore, Bipana Budha, Nar K. Gurung, Jason T. Sawyer

**Affiliations:** 1 Department of Animal Sciences, Auburn University, Auburn, AL 36849, USA; 2 Department of Agricultural and Environmental Sciences, Tuskegee University, Tuskegee, AL 36088, USA

**Keywords:** color, objective tenderness, storage-life, small ruminants

## Abstract

Reducing input costs, especially feed ingredients, remains a priority for production agriculture. Identifying and selecting nutritionally dense ingredients is vital to maximize animal performance. Objectives of the current study were to evaluate the impact of biochar supplementation on goat carcass characteristics and fresh meat quality. Goats (N = 36) were allocated to a diet concentration formulated with or without (Control, Low, Medium, or High g/kg) biochar. After 60 days of feeding, goats were harvested, and carcass measurements were collected. Subprimals from the leg were fabricated into steaks for laboratory analysis of surface color, cook loss, and instrumental tenderness. Biochar supplementation did not alter organ weights (*p* = 0.0614), dressing percentage (*p* = 0.8139), loin eye area (*p* = 0.9570), or tenderness (*p* = 0.0144). However, marbling scores were lower in goats fed at the medium biochar supplementation rate (*p* = 0.0114) and high supplementation (*p* = 0.0102) compared to the control. An interaction between storage day and biochar supplementation was recorded for instrumental surface color lightness (L*; *p* = 0.0016), redness (a*; *p* = 0.0547), hue angle (*p* = 0.0313), and red-to-brown (*p* = 0.0591). Steaks from the 0.052% supplementation group exhibited greater (*p* = 0.0003) redness (a*) during a 7-day refrigerated display and increased chroma values (*p* < 0.0001). Storage duration influenced all color traits, with steak surface discoloration increasing as storage time increased (*p* < 0.0001). Results conclude that biochar supplementation does not negatively impact all carcass quality or tenderness traits, but may influence fat deposition and improve meat color stability.

## Simple Summary

Biochar is a carbon-rich material manufactured by heating plant material with little to no oxygen. Common uses of biochar have been focused on soil management; recent interest has suggested using biochar as a livestock feed additive. It has been reported that biochar may improve digestion, influence fat deposition, and potentially reduce methane emissions. However, there is limited research throughout the literature on the effect biochar has on meat and carcass quality, specifically goats. Goats efficiently produce lean meat from forage rich diets and demand for goat meat in the United States is increasing. In this study, goats were fed diets containing varying levels of biochar to identify any changes to carcass characteristics and meat quality attributes. Greater amounts of supplementation reduced intramuscular marbling, while moderate rates of diet inclusion altered surface redness values and improved the stability of fresh meat throughout storage. Results suggest that biochar may be effectively used as a feed additive without negatively impacting carcass quality of goats.

## 1. Introduction

In recent years, incorporating dietary additives in ruminant nutrition has become more popular to improve animal performance, carcass quality and the overall carcass value [1]. Biochar is an additive that has emerged due to its adsorptive capacity, influence on rumen fermentation and potential role for decreasing methane emissions [2]. It is produced through pyrolysis from types of biomass in a low-to-no oxygen thermal process [3]. Since 2010, biochar has been increasingly used as a feed supplement in cattle, pigs, goats, and fish diet formulations to improve animal health and feed efficiency [3]. Biochar is traditionally considered for use in soil management but has been recently proposed as a supplemental ingredient for use in livestock diets [3].

In many regions globally, goats represent an important livestock species due to their adaptability, lean carcasses, and unique sensory characteristics of their meat [4]. Within the USA, demand for goat meat has increased due to growth in ethnic diversity [4,5]. Goat production in the USA has increased by about one-third over the past decade because of their economic value [4]. Goats are known for feed efficiency, turning low-quality forages into quality meat, milk, and even hides. As ethnic diversity within the USA increased, the market for goat products such as meat, milk, and cheese also increased [5]. Furthermore, with a general population interest in sustainable home practices, there has been an increased interest in Americans producing their own food and using small ruminants such as goats to establish a quality protein. A small herd of goats is an agricultural opportunity that requires minimal space and time to achieve self-sufficiency [4].

Given the growing market of goat production in the United States, identifying optimal feeding strategies to enhance agriculture productivity and meat product quality is important. Nutritional supplementation can be used effectively to improve carcass traits, extend shelf life of fresh meat, and maintain sensory characteristics that niche consumers demand [6]. Replacing a portion of conventional feed ingredients with biochar has become increasingly popular due to the benefits in production and sustainability efforts [2]. Biochar use has been shown to bind toxins, ammonia, and other fermentation byproduct, resulting in greater rumen function and overall improved animal health [2,3]. More specifically, biochar has reduced methane emissions in ruminants [2]. Use of biochar has been widely studied throughout the poultry industry, with reports detailing improved growth performance, nutrient utilization, and meat quality traits [7,8]. Research on biochar supplementation has been extensive in poultry production, particularly in broiler performance [7]. Only a few studies have primarily focused on the use of biochar in the diet of cattle and sheep [9,10,11]. Unfortunately, using biochar in goats remains unexplored. With goats growing role in the United States, investigating biochar supplementation is beneficial to determine whether it can provide a benefit in other ruminant species extending beyond the goat carcass and meat quality traits.

A gap in research studies throughout the literature on ruminant animal performance using biochar supplementation exists. Therefore, the objective of this study was to evaluate the effects of biochar supplementation on goat carcass characteristics, color stability, and cooked characteristics of goat steaks.

## 2. Materials and Methods

### 2.1. Raw Materials

Spanish × Boer goats (N = 36), 10 to 11 months of age with a mean body weight of 36.56 kg (±3.7 kg) were cared for and fed by Tuskegee University (Tuskegee, AL, USA), according to institutional animal care and use guidelines (PRN:R11-2024-13). Goats were fed a 16% crude protein commercially available pelleted ration (Alabama Farmers’ Cooperative Inc., Notasulga, AL, USA) ad libitum. Nutritional composition of commercial diet is provided in Table 1.

Basal diets were supplemented with varying levels of biochar on a dry-matter basis as outlined in Table 2. Supplementation rates are described as control (no biochar), low (2 g/kg of feed), medium (4 g/kg of feed), and high (6 g/kg of feed). Diets and biochar were analyzed using a contract laboratory (Edenridge Labs Ltd., Millersburg, OH, USA). Goats were housed indoors in individual pens (1.1 m × 1.2 m) with plastic-coated, metal-slated floors. Goats were fed individually to allow for measurement of feed intake on a per-animal basis; feed offered and refusals were weighed daily for each goat to assist in determining the individual dry matter intake throughout the experimental period. Dietary inclusion of biochar was designed to evaluate linear and quadratic dose-dependent effects on the animal performance and health. Biochar was produced from Douglas fir (*Pseudotsuga menziesii*) biomass using a bench-scale slow pyrolysis reactor [12], and incorporated into pelleted feed on a dry matter (DM) basis. Following a 60-day feeding period, goats were transported to Auburn University for harvest. Goats were harvested at the Lambert-Powell Meats Laboratory using USDA humane slaughter standards under simulated commercial conditions. During harvest, organ weights were collected and individually weighed using an analytical scale (HD-60KCWP, A&D Company Ltd., Toshima City, Tokyo, Japan). Carcasses were chilled after harvest at 2.0 ± 1.0 °C for 12 h. Chilled carcasses were re-weighed and evaluated for carcass rating and fat cover [13]. Carcasses were split between the 12th and 13th ribs for carcass measurements of backfat thickness, loin eye area, marbling score, and body wall thickness by university-trained personnel. Backfat thickness and body wall thickness were measured using a stainless-steel ruler to the nearest cm [13]. Loin eye area was measured with a grid to the nearest cm^2^. Dressing percentage was calculated using the following formula: ((Live Animal Weight–Hot Carcass Weight)/(Live Animal Weight) × 100)). Cooler shrink was calculated with the following formula: (Hot Carcass Weight–Chilled Carcass Weight) ÷ (Hot Carcass Weight) × 100)). Carcasses were fabricated according to USDA Institutional Meat Purchase Specifications for Fresh Goat meat [13]. During fabrication of the carcass, two steaks from each leg were cut into 2.54 cm thick steaks using a bandsaw (Model 334, Biro Manufacturing Company, Marblehead, OH, USA), labeled, and packaged for analysis.

### 2.2. Packaging and Refrigerated-Display Conditions

Steaks assigned to instrumental fresh color were placed on a foam tray (2S, GENPAK, Charlotte, NC, USA) with an absorbent pad (DRI-LOC AC-50, Novipax, Oak Brook, IL, USA) and wrapped by hand with a polyvinyl chloride film (O^2^ transmission rate = 14,000 cc O^2^/m/24 h/atm). Packaged steaks were stored under constant lighting 2297 lux (TOM-600-12-V4-3, Phillips Xitanium, Seoul, Republic of Korea) at 3.0 °C ± 1.5 °C throughout the 7-day storage period. Remaining steaks were assigned to cook loss and shear force (n = 18/treatment). Steaks allocated to cook loss and objective tenderness were identified and vacuum packaged individually in 20.32 cm × 25.40 cm (l × w) 3 mil (nylon/polyethylene) pouches (Prime Source, St. Louis, MO, USA), with an oxygen transmission rate (7.5 cc/100 square inches/24 h of atm). Vacuum packages were sealed using a double chamber vacuum packaging machine (Model UV2100-C, Koch Equipment LLC, Kansas City, MO, USA) and stored refrigerated at 3.0 °C ± 1.5 °C in the absence of light until analysis was completed (24 h).

### 2.3. Objective Fresh Color

Surface color of each steak (n = 18/treatment) was measured using a HunterLab EZ colorimeter (Model 45/0 LAV, Hunter Associates Laboratory Inc., Reston, WV, USA) on each day of the refrigerated storage. Color readings were determined from the mean of three readings of each steak through the packaging film using illuminant A, a 10° observer and a 31.88 mm aperture, according to the American Meat Science Association guidelines [14]. Prior to scanning, the colorimeter was calibrated using a black and white tile per the manufacturer’s guidelines. Calculated values of hue angle (°) were determined by [tan − 1 (b*/a)], and chroma (C*) was calculated using the following equation: (√(b*)^2^ + (a*)^2^). Reflectance values from 400 to 700 nm were used to record surface color changes from red to brown using the reflectance ratio of 630 nm/580 nm. Relative values for deoxymyoglobin, metmyoglobin, and oxymyoglobin were determined using formulas provided by the AMSA Meat Color Measurement Guidelines [14]. Deoxymyoglobin (DMb) was determined using the equation (%DMb = {2.375 × [1 − (A473 − A730)/(A525 − A730)]} × 100). Metmyoglobin (MMb) was calculated using the equation (%MMb = {1.395 − [(A572 − A730)/(A525 − A730)]} × 100). Oxymyoglobin (OMb) was calculated using the equation [%OMb = 100 − (%MMb − %DMb)].

### 2.4. Cooked Characteristics

Prior to cooking, each steak was removed from the package, blotted dry with a paper towel, and weighed (Model PB2003-2, Mettler Toledo, Columbus, OH, USA) (n = 18/treatment). Steaks were cooked using a convection oven preheated to 177 °C to an internal temperature of 71 °C. Internal temperature of each steak was recorded in the geometric center using a hand-held digital thermometer (Therma-K Plus, American Fork, UT, USA). Steaks were removed from the oven, cooled to room temperature, and re-weighed. Cook loss was determined using the following equation: ((Raw Weight–Cooked Weight)/Raw Weight) × 100)). Six cores were removed by a hand-held corer parallel to the muscle fiber of each steak and sheared once, using a texture analyzer (Model TA.XT.Plus 100C, Texture Technologies Corp., New York, NY, USA) equipped with a 30 kg load cell and a crosshead speed of 2.00 mm/s. Peak force values for each core were averaged to calculate the average peak force (N) value.

### 2.5. Statistical Analysis

Data was analyzed using the GLIMMIX procedures of SAS (version 9.2; SAS Inst., Cary, NC, USA). Least square means were computed for all dependent variables including organ weights, carcass traits, and meat quality traits. Fixed effects of treatment were included in the model with goat serving as the experimental unit. A repeated measure (day) was included in the model for instrumental surface color variables. Orthogonal contrasts were calculated to compare biochar inclusion dietary treatments to the control diet. Significant differences were declared at *p* ≤ 0.05, and least square means were separated using pairwise *t*-tests (PDIFF option).

## 3. Results and Discussion

### 3.1. Organ Weight and Carcass Characteristics

Visceral organ weight can reflect metabolic function and overall health of an animal. In the current study, statistical differences were not recorded for organ weights of the heart (*p* = 0.0739), liver (*p* = 0.0614), kidney (*p* = 0.3179), or spleen (*p* = 0.1892) measured during goat harvest (Table 3), although a numerical trend occurred for heart and liver weights as supplementation of biochar increased. It is plausible a declining trend in organ weight could reflect a shift in nutrient allocation toward muscle or fat deposition, but, certainly, additional research is needed to identify the influence of ruminal variables on physiological characteristics. Organ weights were analyzed using orthogonal contrasts and confirmed that the use of biochar supplementation compared to the control diets did not significantly alter heart (*p* = 0.5906), kidney (*p* = 0.4340), liver (*p* = 0.0829), or spleen (*p* = 0.1892) weights. These results do not agree with the previous studies reporting high doses or long-term feeding of biochar can result in measurable changes to organ weights. Incorporating corncob biochar into a sheep’s diet significantly affected the weight of the liver, intestines and pancreas, compared to the control [9]. It is important to note that although there was only a small variation in the organ weights, all organs fall within the normal weight variability [15]. Overall, biochar supplementation did not adversely impact visceral organ development.

Carcass characteristics are important indicators of production efficiency and market value (Table 4). In this study, most carcass values were not affected by biochar supplementation in the diet, apart from one major carcass characteristic—marbling. Intramuscular fat deposition was significantly influenced (*p* = 0.0250) by the diet supplementation of biochar. Goats allocated to the medium- and high-supplementation groups had less intramuscular fat in the loin eye (lower marbling scores) compared to goats consuming the control or low-supplementation diets. A reduced marbling score suggests that biochar can reduce the deposition of intramuscular fat at higher inclusion rates. It is plausible that alterations in rumen fermentation and energy partitioning caused carcasses to be leaner [10]. Interestingly, contrasts for carcass traits, which indicated lighter hot carcass (*p* = 0.0264) and chilled carcass weights, (*p* = 0.0224) were evident in supplemented goats compared to carcass weights of goats consuming the control diet. Dressing percentage, loin eye area, fat thickness, and cooler shrink were unaffected by consumption of biochar. Limited changes to carcass characteristics suggest that overall carcass quality and yield were maintained regardless of the variation in supplementation. These findings align with the previous literature where biochar supplementation did not compromise carcass yield [11]. Biochar supplementation could, however, influence the pattern of fat deposition resulting in a lower marbling score.

### 3.2. Instrumental Surface Color

**Figure 1:**
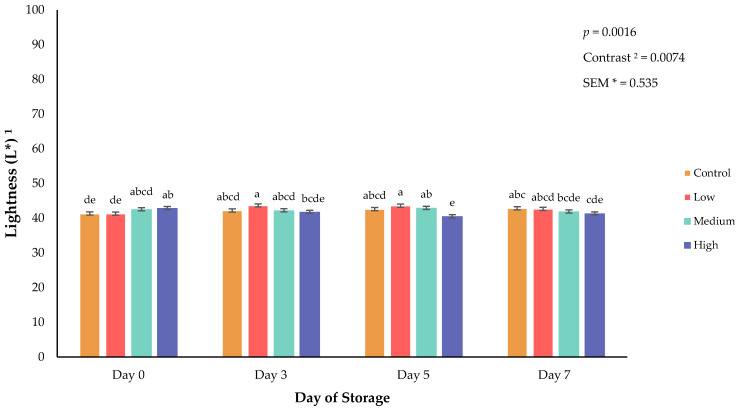
Interaction of biochar supplementation treatment × day of storage on lightness (L*) of fresh goat meat. ^a–e^ Means lacking common superscripts differ (*p* < 0.05). * SEM, standard error of the mean. ^1^ Lightness (L*) values are a measure of darkness to lightness where 100 is white, and 0 is black. ^2^ Orthogonal contrasts: Biochar supplementation vs. control.

**Figure 2:**
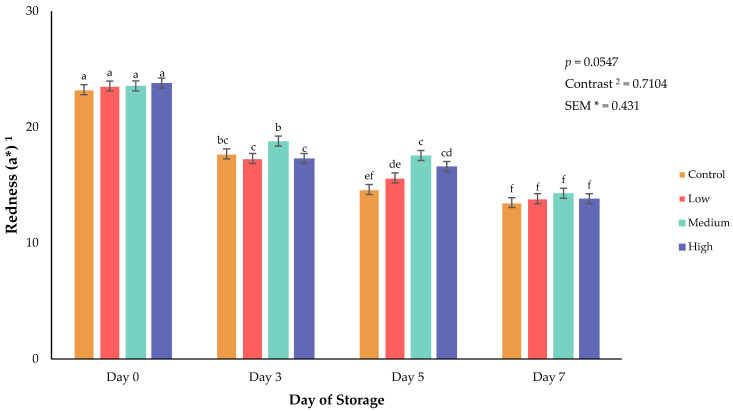
Interaction of biochar supplementation treatment × day of storage on redness (a*) of fresh goat meat. ^a–f^ Means lacking common superscripts differ (*p* < 0.05). * SEM, standard error of the mean. ^1^ Redness (a*) values are a measure of redness where larger value indicates a redder color. ^2^ Orthogonal contrasts: Biochar supplementation vs. control.

**Figure 3:**
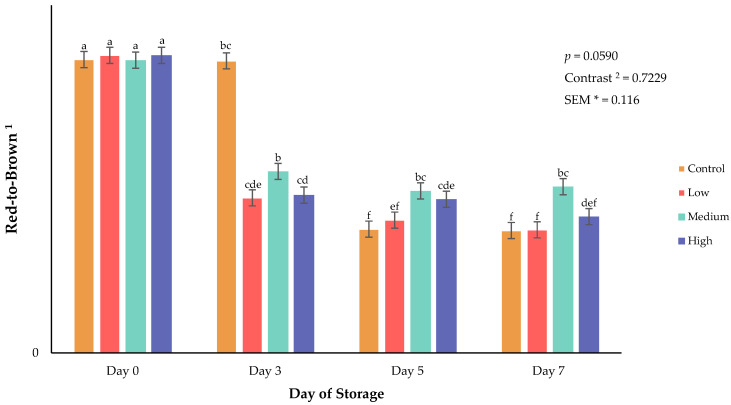
Interaction of biochar supplementation treatment × day of storage on hue angle of fresh goat meat. ^a–f^ Means lacking common superscripts differ (*p* < 0.05). * SEM, standard error of the mean. ^1^ RTB is the reflectance ratio of 630 nm ÷ 580 nm and represents a change in the color of red to brown (a larger value indicates a redder color). ^2^ Orthogonal contrasts: Biochar supplementation vs. control.

**Figure 4:**
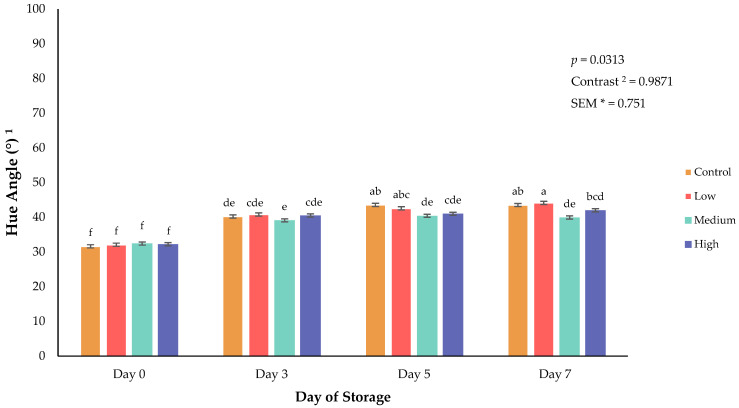
Interaction of biochar supplementation treatment × day of storage on red-to-brown of fresh goat meat. ^a–f^ Means lacking common superscripts differ (*p* < 0.05). * SEM, standard error of the mean. ^1^ Hue angle (°) represents the change in color from the true red axis (a larger number indicates a greater shift from red to yellow). ^2^ Orthogonal contrasts: Biochar supplementation vs. control.

Surface color of fresh meat can greatly influence consumer purchasing decisions [16]. Beyond purchasing at a retail level, surface color can impact a consumer’s appeal to a product or perceived quality [17]. Specifically, lightness (L*) is a surface color attribute that influences consumer perception of meat brightness and overall quality [16,18]. In the current study, a significant interaction between biochar supplementation and refrigerated storage day of retail steaks for lightness (*p* = 0.0025; Figure 1), redness (*p* = 0.0547; Figure 2), red-to-brown (*p* = 0.0591; Figure 3), and hue angle (*p* = 0.0313; Figure 4) was observed. Additionally, orthogonal contrasts reveal that goats consuming biochar supplementation exhibited greater surface color lightness values, indicative of a lighter color than fresh meat from goats consuming the control diet (*p* = 0.0074). Throughout refrigerated storage of packaged retail steaks, all treatments displayed increased surface lightness by day 3, indicative of color blooming followed by a decline through the end of the 7-day storage period. This decline may be the result of a reduction in moisture occurring during storage, oxidation of lipids, or changes in red or white muscle fibers [19]. Redness values support the changes in surface lightness as steak redness was greatest on day 0 with steaks from goats consuming the medium supplementation diet, maintaining the greatest redness values throughout the 7-day storage duration. Hue angle differed among treatments across storage duration, with goats consuming the control and low-biochar supplementation diets having a greater shift from red to yellow after 3 days of refrigerated storage. Considering the limitations of supporting evidence on meat surface color in the previous study, it was concluded that greater dietary biochar supplementation can increase redness values of poultry meat [8]. By day 7 of storage in the current study, hue angle increased in all dietary treatment groups, indicating a shift toward a brown surface discoloration. Red-to-brown ratios support the current findings as all treatments exhibited lower RTB values by the end of the storage duration, with steaks from the goats consuming the medium concentration of dietary biochar having the greatest values on day 5 and 7 of storage. These trends align with established patterns of meat color degradation during aerobic storage [20].

The main effects of dietary treatment were observed for chroma and yellowness (Table 5). Steaks from goats consuming the medium concentration of biochar supplementation group had greater chroma values compared to all other treatments, while yellowness values were less for steaks from goats consuming the control treatment and greatest for steaks from medium supplementation carcasses. It is well known that increased storage time can affect the color of fresh meat products [20].

Storage duration decreased both chroma and yellowness with declining values after day 0, resulting in a less-vivid product and gradual discoloration (Table 6). These results suggest feeding biochar at the medium dietary inclusion rate, described in Table 1, could enhance surface color attributes of fresh-goat retail meat cuts and potentially increase consumer appeal. Results from the current study are supported by the previous findings with dietary interventions being linked to alterations in the color of fresh meat products [21,22]. In this study, storage duration significantly influenced all surface color attributes (*p* < 0.0001).

Myoglobin drives fresh meat color and plays a critical role in determining visual appeal to consumers [20]. In the current study, main effects of the treatment revealed that biochar supplementation significantly influenced relative values of metmyoglobin (*p* < 0.0001) and oxymyoglobin (*p* < 0.0001), while deoxymyglobin was unaffected (*p* = 0.8712) using dietary biochar (Table 7). Contrast analysis indicates there was no overall difference between biochar supplementation and control groups for the relative values of deoxymyoglobin (*p* = 0.934), metmyoglobin (*p* = 0.3101), or oxymyoglobin (*p* = 0.3945). Goats consuming the medium-supplementation level resulted in the least relative metmyoglobin percentage and the greatest relative oxymyoglobin percentage, when compared to the steaks from control and low-dietary supplementation level. Results of relative myoglobin values suggest that medium concentration of dietary biochar inclusion may enhance red color stability and reduce browning due to oxidation.

Storage day affected all measured relative forms of myoglobin (*p* < 0.0001) as presented in Table 8. Oxymyoglobin decreased steadily throughout the storage period. This decline coincided with an increase in metmyoglobin, representing an oxidative discoloration over time. These changes are consistent with the previous literature of meat discoloration patterns in red meats when oxidation processes begin to convert oxymyoglobin to metmyoglobin, resulting in a reduced consumer appeal [23]. Previous and current results concluded that an oxidative shift is inevitable during storage regardless of packaging systems. It is well known throughout the literature that using oxygen-permeable package materials will cause surface color discoloration to occur more rapidly. However, the inclusion of a dietary supplement such as biochar suggests, based on the current surface color results, that it is plausible that the oxidation and surface color deterioration can be decreased.

### 3.3. Cook Loss and Shear Force

Tenderness of a product is a key determinant of consumer eating satisfaction. It is well known that cooking loss is often associated with the tenderness of a product based on the amount of fat and moisture lost during the cooking process. In the results of the current study, biochar supplementation did not affect cook loss (*p* = 0.4077). However, objective tenderness values were affected by supplementation (*p* = 0.0144), with control and low supplementation producing more tender products compared to the medium- and high-supplementation groups (Table 9). It is important to note that a decrease was measured in the amount of cook loss for the medium- and high-supplementation groups, but the difference was not significant. Moreover, shear force values were slightly greater in the high-biochar-supplementation-diet-fed goats, reflecting the reduction in tenderness as biochar inclusion rate increase. In contrast, previous research results while extremely limited report an improvement in juiciness, tenderness, and water-holding capacity in the meat of ducks that were fed with a biochar supplementation [24]. It has been reported previously that biochar is a non-reactive substance, and it should not affect most meat quality parameters [25]. Calculated orthogonal contrasts conclude that no overall difference between biochar-supplemented and control-fed goats for cook loss (*p* = 0.3025) or shear force values (*p* = 0.0626) occurred. Current findings further support that biochar supplementation does not affect cooking yield; supplementation may impact tenderness of a product which is crucial for consumer satisfaction.

## 4. Conclusions

Biochar supplementation used as a feed additive in goat diets did not alter calculated carcass yield, organ weights, cooking loss or objective tenderness. However, biochar supplementation exceeding 4 g/kg of the diet caused a reduction in marbling scores. Surprisingly, 4 g/kg of biochar improved surface color redness and delayed discoloration during refrigerated storage conditions for goat steaks. These results indicate that biochar should be considered in the diet formulation of small ruminants such as goats. Future studies are most definitely needed to provide greater results on biochar supplementation effects in other livestock species, as well as consumer acceptability and economic considerations of using byproduct feed ingredients such as biochar in production systems.

## Figures and Tables

**Table 1:** Commercial diet formulation.

Nutrient	Percentage (%)
Dry matter	88 to 90%
Moisture	10 to 12%
Crude Protein (min)	16.00%
Crude Fat (min)	3.00%
Crude Fiber (max)	12.00%
Grain By-Prod, Grains, Roughage, Plant Protein, CaCO_3_, Salt, NH_4_Cl, Vit A, Vit D_3_, Vit E, Se, Fe, Mn, Zn, Cu, I, Co, Min Oil	% not disclosed

**Table 2:** Nutritional composition of goat diets with varying levels of Biochar.

Nutrient Analysis	Dietary Concentration of Biochar %				Biochar
	Control 0 g Biochar/kg	Low 2 g Biochar/kg	Medium 4 g Biochar/kg	High 6 g Biochar/kg	
Moisture (%)	10.59	10.77	11.32	11.64	13.29
Dry Matter (%)	89.41	89.23	88.68	88.36	86.71
Crude Protein (%)	16.67	15.17	15.89	13.41	2.86
Lignin (%)	4.52	4.76	5.48	5.01	55.83
Acid Detergent Fiber (%)	13.14	12.56	14.76	12.41	60.40
Neutral Detergent Fiber (%)	23.58	26.83	27.55	22.37	62.77
Crude Fat (%)	4.01	4.08	3.65	3.44	1.69
Ash (%)	7.63	7.43	9.16	7.34	9.56
TDN	73.81	72.75	69.50	73.20	25.89
Lignin Insoluble Ash (%)	1.29	0.65	1.90	0.87	4.58
Calcium (Ca, %)	1.10	1.07	1.11	1.07	1.02
Phosphorus (P, %)	0.61	0.60	0.60	0.56	0.06
Magnesium (Mg, %)	0.30	0.29	0.31	0.30	0.18
Potassium (K, %)	1.02	1.00	1.04	1.03	0.19
Sulfur (S, %)	0.19	0.20	0.19	0.20	0.03
Sodium (Na, %)	0.341	0.316	0.305	0.299	0.109
Copper (Cu, ppm)	7	10	14	9	6
Manganese (Mn, ppm)	101	100	99	98	636
Zinc (Zn, ppm)	215	256	200	257	6
Iron (Fe, ppm)	123	136	146	138	334

Values are expressed on a dry matter (DM) basis, except for moisture and DM, which are reported on an as-fed basis.

**Table 3:** Influence of biochar supplementation on goat organ weight.

	DIETARY TREATMENT						
	Control	Low	Medium	High	SEM *	*p*-Value	Contrast ^1^
Heart (g)	166.14	140.47	133.94	140.47	9.11	0.0739	0.5906
Liver (g)	505.57	434.03	427.03	398.30	27.69	0.0614	0.0829
Kidney (g)	102.72	95.67	139.46	95.15	19.08	0.3179	0.4340
Spleen (g)	57.91	55.51	65.47	48.83	5.27	0.1892	0.1892

* SEM—standard error of the mean. ^1^ Orthogonal contrast: biochar supplementation vs. control.

**Table 4:** Influence of biochar supplementation on goat carcass characteristics.

	DIETARY TREATMENT						
	Control	Low	Medium	High	SEM *	*p*-Value	Contrast ^1^
Hot Carcass Weight	16.53	15.27	16.04	14.26	0.622	0.0779	0.0264
Back Fat Thickness (cm^2^)	0.18	0.23	0.15	0.14	0.037	0.3809	0.4545
Cold Carcass Weight (kg)	16.12	14.97	15.76	13.95	0.594	0.0717	0.0224
Fat Cover	1.83	1.78	1.63	1.44	0.136	0.1999	0.2200
Carcass Rating	2.16	2.67	2.43	2.89	0.208	0.1079	0.3447
Cooler Shrink (%)	2.45	2.02	1.76	2.21	0.909	0.9616	0.9004
Dressing Percentage (%)	41.57	43.12	42.49	42.65	1.156	0.8139	0.8468
Loin Area (cm^2^)	10.82	10.39	10.56	10.68	0.563	0.9570	0.9888
Marbling Score	288.89 ^a^	250.00 ^a^	187.50 ^b^	155.89 ^b^	25.856	0.0250	0.0120
Body Wall Thickness	1.27	1.19	1.25	1.16	0.084	0.7554	0.0807

* SEM—standard error of the mean. ^a,b^ Mean values within the main effect of treatment lacking common superscripts differ (*p* < 0.05). ^1^ Orthogonal contrast: biochar supple-mentation vs. control.

**Table 5:** Influence of biochar supplementation on fresh goat meat surface color.

	DIETARY TREATMENT						
	Control	Low	Medium	High	SEM *	*p*-Value	Contrast ^1^
Yellowness (b*) ^2^	14.15 ^c^	14.45 ^ab^	14.82 ^a^	14.60 ^b^	0.157	0.0240	0.4899
Chroma ^3^	22.89 ^c^	23.36 ^bc^	24.68 ^a^	23.56 ^b^	0.136	<0.0001	0.5953

* SEM—standard error of the mean. ^a–c^ Mean values within the main effect of treatment lacking common superscripts differ (*p* < 0.05). ^1^ Orthogonal contrast: Biochar supplementation vs. control. ^2^ Yellowness (b*) values are a measure of yellowness where larger value indicates a more yellow color. ^3^ Chroma is a measure of total color (a larger number indicates a more vivid color).

**Table 6:** Day of storage impact on fresh goat meat surface color.

	DAY OF STORAGE									
	0	1	2	3	4	5	6	7	SEM *	*p*-Value
Yellowness (b*) ^1^	14.97 ^b^	15.88 ^a^	15.88 ^a^	14.94 ^b^	14.48 ^c^	14.29 ^cd^	13.96 ^de^	13.85 ^e^	0.134	<0.0001
Chroma ^2^	27.90 ^a^	26.07 ^b^	26.07 ^b^	23.24 ^c^	22.32 ^d^	21.57 ^e^	21.03 ^ef^	20.80 ^f^	0.242	<0.0001

* SEM—standard error of the mean. ^a–f^ Mean values within the main effect of treatment lacking common superscripts differ (*p* < 0.05). ^1^ Yellowness (b*) values are a measure of yellowness where larger value indicates a more yellow color. ^2^ Chroma is a measure of total color (a larger number indicates a more vivid color).

**Table 7:** Day of storage impact on fresh goat meat myoglobin values.

	DAY OF STORAGE									
	0	1	2	3	4	5	6	7	SEM *	*p*-Value
Metmyoglobin (%)	23.53 ^e^	32.34 ^d^	32.34 ^d^	40.74 ^c^	42.59 ^b^	44.91 ^a^	45.82 ^a^	45.93 ^a^	0.598	<0.0001
Oxymyoglobin (%)	55.89 ^b^	55.22 ^a^	55.22 ^a^	47.27 ^c^	45.11 ^d^	42.24 ^e^	41.16 ^ef^	40.16 ^f^	0.669	<0.0001
Deoxymyoglobin (%)	23.58 ^a^	12.43 ^c^	12.43 ^c^	11.99 ^c^	12.29 ^c^	12.85 ^bc^	13.02 ^bc^	13.90 ^b^	0.456	<0.0001

* SEM—standard error of the mean. ^a–f^ Mean values within each row lacking common superscripts differ (*p* < 0.05). Calculated percentages of metmyoglobin, deoxymyoglobin, and oxymyoglobin using relative spectral values.

**Table 8:** Treatment effect on fresh goat meat myoglobin values.

	DIETARY TREATMENT						
	Control	Low	Medium	High	SEM *	*p*-Value	Contrast ^1^
Metmyoglobin (%)	39.81 ^a^	39.45 ^a^	35.95 ^b^	38.89 ^a^	0.423	<0.0001	0.3101
Oxymyoglobin (%)	46.03 ^b^	46.34 ^b^	50.19 ^a^	47.06 ^b^	0.473	<0.0001	0.3945
Deoxymyoglobin (%)	14.16	14.20	14.85	14.04	0.323	0.8712	0.9344

* SEM—standard error of the mean. ^a,b^ Mean values within each row lacking common superscripts differ (*p* < 0.05). Calculated percentages of metmyoglobin, deoxymyoglobin, and oxymyoglobin using relative spectral values. ^1^ Orthogonal contrast: Biochar supplementation vs. control.

**Table 9:** Influence of biochar supplementation on fresh goat meat cooking loss and texture.

	DIETARY TREATMENT						
	Control	Low	Medium	High	SEM *	*p*-Value	Contrast ^1^
Cook Loss (%)	30.23	28.78	25.74	25.50	2.29	0.3848	0.3025
Shear Force (N)	37.84 ^b^	38.04 ^b^	39.80 ^ab^	43.85 ^a^	1.52	0.0144	0.0626

* SEM—standard error of the mean. ^a,b^ Mean values within each row lacking common superscripts differ (*p* < 0.05). ^1^ Orthogonal contrast: Biochar supplementation vs. control.

## Data Availability

Original contributions presented in this study are included in the article. Further inquiries can be directed to the corresponding author.

## Abbreviations

The following abbreviations are used in this manuscript:

SEM	Standard error of the mean
g	Gram
kg	Kilogram
DM	Dry matter
